# Unchanged triclabendazole kinetics after co-administration with ivermectin and methimazole: failure of its therapeutic activity against triclabendazole-resistant liver flukes

**DOI:** 10.1186/1746-6148-6-8

**Published:** 2010-02-03

**Authors:** Laura Ceballos, Laura Moreno, Luis Alvarez, Laura Shaw, Ian Fairweather, Carlos Lanusse

**Affiliations:** 1 Laboratorio de Farmacología, Facultad de Ciencias Veterinarias, Universidad Nacional del Centro de la Provincia de Buenos Aires (UNCPBA), Campus Universitario, 7000, Tandil, Argentina; 2 Consejo Nacional de Investigaciones Científicas y Técnicas (CONICET), Argentina; 3 School of Biological Sciences, Medical Biology Centre, The Queen's University of Belfast, Belfast, Northern Ireland, BT9 7BL, UK

## Abstract

**Background:**

The reduced drug accumulation based on enhanced drug efflux and metabolic capacity, identified in triclabendazole (TCBZ)-resistant *Fasciola hepatica *may contribute to the development of resistance to TCBZ. The aim of this work was to evaluate the pharmacokinetics and clinical efficacy of TCBZ administered alone or co-administered with ivermectin (IVM, efflux modulator) and methimazole (MTZ, metabolic inhibitor) in TCBZ-resistant *F. hepatica*-parasitized sheep. Sheep infected with TCBZ-resistant *F. hepatica *(Sligo isolate) were divided into three groups (n = 4): untreated control, TCBZ-treated (i.r. at 10 mg/kg) and TCBZ+IVM+MTZ treated sheep (10 i.r., 0.2 s.c. and 1.5 i.m. mg/kg, respectively). Plasma samples were collected and analysed by HPLC. In the clinical efficacy study, the animals were sacrificed at 15 days post-treatment to evaluate the comparative efficacy against TCBZ-resistant *F. hepatica*.

**Results:**

The presence of IVM and MTZ did not affect the plasma disposition kinetics of TCBZ metabolites after the i.r. administration of TCBZ. The AUC value of TCBZ.SO obtained after TCBZ administration (653.9 ± 140.6 μg.h/ml) was similar to that obtained after TCBZ co-administered with IVM and MTZ (650.7 ± 122.8 μg.h/ml). Efficacy values of 56 and 38% were observed for TCBZ alone and for the combined treatment, respectively. No statistical differences (P > 0.05) were observed in fluke counts between treated groups and untreated control, which confirm the resistant status of the Sligo isolate.

**Conclusions:**

The presence of IVM and MTZ did not affect the disposition kinetics of TCBZ and its metabolites. Thus, the combined drug treatment did not reverse the poor efficacy of TCBZ against TCBZ-resistant *F. hepatica*.

## Background

Triclabendazole (TCBZ, 6-chloro-5(2-3 dichlorophenoxy)-2-methyl thio-benzimidazole), an halogenated benzimidazole (BZD) thiol derivative, shows high efficacy against both the immature and mature stages of *Fasciola hepatica *in sheep and cattle, which is a differential feature compared to other available trematodicidal drugs [1]. As a consequence of its excellent activity against the liver fluke, it has been extensively used and this has inevitably promoted the selection of TCBZ-resistant populations, which is now a worrying problem in several areas of the world [2,3].

Parasites have several possible strategies to achieve drug resistance, including changes in the target molecule, in drug uptake/efflux mechanisms and in drug metabolism [4]. At least two mechanisms appear to be implicated in TCBZ resistance in *F. hepatica*: increased drug efflux and enhanced oxidative metabolism [5-7]. TCBZ and its sulphoxide metabolite (TCBZ.SO) are both substrates of P-glycoprotein (Pgp) [8]. Over-expression of Pgp has been implicated in the resistance to macrocyclic lactones (ivermectin (IVM), moxidectin (MXD)) [9,10], closantel and BZDs in nematodes [11]), although the exact nature of the role has yet to be established [12]. Different *ex vivo *experiments support the hypothesis of the involvement of Pgp over-expression in the resistance of *F. hepatica *to TCBZ. Higher levels of TCBZ and TCBZ.SO were observed within TCBZ-resistant flukes when drug efflux from the parasite was decreased by IVM [7], a well recognized Pgp substrate/inhibitor [9,13]. It has been demonstrated that TCBZ and its main metabolites, TCBZ.SO and TCBZ-sulphone (TCBZ.SO_2_) may induce tegumental damage in liver flukes [14]. Additionally, an increased oxidative metabolic capacity has been described as complementary TCBZ resistance mechanism in *F. hepatica *[5,6]. In fact, co-incubation of TCBZ or TCBZ.SO with methimazole (MTZ), a flavin monooxygenase (FMO) enzymatic system inhibitor, lead to more severe surface morphological changes in TCBZ-resistant *F. hepatica*, compared to that observed after incubation with TCBZ or TCBZ.SO alone [15].

The interaction between co-administered drugs may induce changes in the pharmacokinetic behaviour of either molecule. Increased albendazole sulphoxide plasma concentrations in lambs after co-administration of albendazole (intraruminally, i.r.) with IVM (subcutaneously, s.c.), was previously reported [16]. Similarly, after the co-administration to sheep of IVM and TCBZ by the intravenous (i.v.) route, an enhanced TCBZ.SO plasma concentration was achieved [17]. On the other hand, MTZ inhibition of TCBZ oxidative metabolism by sheep liver microsomes has been reported [18]. However, MTZ did not affect TCBZ disposition kinetics in sheep after the administration of both compounds by the i.v. route [19].

Both modified influx/efflux and enhanced metabolism may account for the development of resistance to TCBZ in *F. hepatica*. As a consequence, it opens up the possibility of modulating drug efflux and metabolism in the TCBZ-resistant fluke, by co-administering TCBZ with MTZ and IVM, with the aim of reversing anthelmintic resistance. Furthermore, *in vivo *drug-drug interaction between these drugs may modify the overall disposition kinetics and pattern of drug distribution of TCBZ to the liver fluke. The aims of the current work were: a) to investigate the potential effect of MTZ and IVM on the plasma concentrations profiles of TCBZ and its metabolites in sheep and b) to study the clinical efficacy of TCBZ alone or when co-administered with MTZ and IVM against TCBZ-resistant *F. hepatica*.

## Methods

### Chemicals

Pure reference standards (97-99%) of TCBZ and its TCBZ.SO and TCBZ.SO_2 _metabolites, were provided by Novartis Animal Health (Basel, Switzerland) (Batch # AMS 215/102, HI-1025/1 and JG-5161/6, respectively). MTZ and IVM were purchased from Sigma-Aldrich Chemical Company (St Louis, USA). The different solvents (HPLC grade) and buffer salt used for sample extraction or chromatographic methods were purchased from Baker Ind. (Phillipsburg, USA).

### Animals and Experimental design

Twelve (12) healthy intact male Corriedale sheep (53.8 ± 2.6 kg) aged 14-16 months and obtained from a farm located in an area free of *F. hepatica *were involved in this trial. Additionally, the absence of liver fluke infection was checked by analysis of *F. hepatica *eggs in faeces, following routine procedures [20]. Animals were housed individually during the experiment and for 20 days before the start of the study. Animals were fed on a commercial balanced concentrate diet. Water was provided *ad libitum*. Animal procedures and management protocols were carried out in accordance with the Animal Welfare Policy (Act 087/02) of the Faculty of Veterinary Medicine, Universidad Nacional del Centro de la Provincia de Buenos Aires (UNCPBA), Tandil, Argentina http://www.vet.unicen.edu.ar and internationally accepted animal welfare guidelines [21].

Animals were each orally infected with eighty (80) metacercariae of a TCBZ-resistant *F. hepatica *isolate, named Sligo. For details of the history of the Sligo isolate, see previous works [5,22,23]. Sixteen weeks after infection, animals were randomly distributed into three experimental groups (n = 4 each): Group I, which represented the untreated control group; Group II, in which animals were treated with TCBZ (Fasinex^®^, Novartis) by the i.r. route at 10 mg/kg dose rate; and Group III, in which animals were simultaneously treated with TCBZ (Fasinex^®^, Novartis) by the i.r. route (10 mg/kg) and IVM (Ivosint^®^, Biogénesis) by the s.c. route (0.2 mg/kg, internal face of the thigh). Additionally, animals in Group III were treated by the intramuscular (i.m.) route (*Semitendinosus *muscle) with MTZ (2.5% aqueous solution) at a dose rate of 1.5 mg/kg [24]. MTZ administration was performed 30 min after TCBZ/IVM treatment. Blood samples (5 ml) were taken by jugular venipunctures into heparinised Vacutainers^®^ tubes (Becton Dickinson, USA) before administration (time 0) and at 1, 3, 6, 9, 12, 18, 24, 30, 36, 48, 72, 96, 120 and 144 h post-treatment. Plasma was separated by centrifugation at 3000 ***g ***for 15 min, placed into plastic tubes and frozen at -20°C until analyzed by high performance liquid chromatography (HPLC).

### Clinical efficacy study

Fifteen days after treatment all animals were stunned and exsanguinated immediately. Adult *F. hepatica *specimens were recovered from the common bile ducts and the gall bladder of each sheep and counted according to the World Association for the Advancement of Veterinary Parasitology (W.A.A.V.P) guidelines [25]. The efficacy of each anthelmintic treatment was determined by the comparison of *F. hepatica *burdens in treated versus untreated animals. The following equation expresses the percent efficacy (*% E*) of a drug treatment against *F. hepatica *(*F.h.*) in a single treatment group (*T*) when compared with an untreated control (*C*).

The geometric mean was used as it most accurately represents the distribution of parasite populations within each group [25].

### Analytical procedures

#### Plasma sample extraction

TCBZ and its metabolites were extracted from plasma as previously described [18]. Samples (1 ml) were spiked with 10 μl of oxibendazole (OBZ) (100 μg/ml), used as internal standard. After addition of 2 ml of acetonitrile, samples were shaken for 20 min (multivortex) and then centrifuged at 2500 ***g ***for 15 min. The supernatants were recovered and evaporated to dryness in a vacuum concentrator (Speed-Vac^®^, Savant, Los Angeles, USA). The dry extracts were reconstituted in 300 μl of mobile phase and an aliquot of 50 μl was injected into the HPLC system.

#### Drug quantification by HPLC analysis

Experimental and fortified plasma samples were analysed by HPLC to determine the concentration of TCBZ and its metabolites following the methodology previously described [18]. The elution from the stationary phase (Selectosil C_18 _column, 5 μm, 250 × 4.6 mm, Phenomenex^®^, CA, USA) was carried out at a flow rate of 1.2 ml/min, using a mixture of acetonitrile/ammonium acetate (0.025 M, pH 6.6) as mobile phase. Fifty μl of each previously extracted sample were injected into a Shimadzu 10 A HPLC System (Kyoto, Japan), using a gradient pump, UV detector set at 300 nm, an autosampler and a controller (Shimadzu Class LC10, Kyoto, Japan). Analytes were identified by the retention times of pure reference standards. Chromatographic retention times were: 4.09 (OBZ), 5.91 (TCBZ.SO), 7.95 (TCBZ.SO_2_) and 10.36 (TCBZ) min. Calibration curves for each analyte were prepared by least squares linear regression analysis, which showed correlation coefficients between 0.995 and 0.998. The absolute recovery of drug analytes from plasma was calculated by comparison of the peak areas from spiked plasma samples with the peak areas resulting from direct injections of standards in mobile phase. Mean absolute recoveries and coefficient of variations (CV) within the concentration range between 0.1 and 25 μg/ml (triplicate determinations) were 89.2% (CV: 6.74%) (TCBZ), 87.1% (CV: 6.03%) (TCBZ.SO) and 90.1% (CV: 4.75%) (TCBZ.SO_2_). Precision (intra- and inter-assay) was determined by analysing replicates of fortified plasma samples (n = 5) with each compound at three different concentrations (0.1, 5 and 10 μg/ml). CV ranged from 2.54 to 14.6%. The limit of quantification (LOQ) was defined as the lowest measured concentration with a CV <20% and accuracy of ±20% and an absolute recovery ≥70%. The LOQ defined for the three molecules assayed was 0.1 μg/ml. Values below LOQ were not included in the pharmacokinetic analysis.

### Pharmacokinetic analysis

The concentration versus time curves for TCBZ metabolites in plasma for individual animals were fitted with the PKSolutions™ computer program/programme (Summit Research Service, Ashland, USA). Pharmacokinetic analysis of the experimental data was performed by non-compartmental analysis. The first order absorption rate constant (k_ab_) or the first order metabolite formation rate constant (k_f_) (h^-1^) were calculated by the residual method [26]. The elimination half life (T1/2el) and absorption (T1/2ab) or metabolite formation (T1/2for) half lives were calculated as ln2/β and ln 2/k, respectively, where ß represent the terminal slope (h^-1^). The observed peak concentration (C_max_) and time to peak concentration (T_max_) were read from the plotted concentration-time curve of each analyte. The area under the concentration time-curve (AUC) was calculated by the trapezoidal rule [26] and further extrapolated to infinity by dividing the last experimental concentration by the terminal slope (β).Statistical moment theory was applied to calculate the mean residence time (MRT) for metabolites in plasma, as follows:

where AUC is as defined previously and AUMC is the area under the curve of the product of time and the plasma drug concentration versus time from zero to infinity [26].

### Statistical analysis of the data

Pharmacokinetic parameters are presented as mean ± SD. The Student' t-test was used for the statistical comparison of the pharmacokinetic data obtained from both treatments groups. AUC, Cmax, MRT, T1/2el and T1/2for values were log-transformed before statistical analysis. Untransformed Tmax values were compared by non-parametric test (Mann-Withney Test). Fluke counts in each experimental group were compared by non parametric test (Kruskal-Wallis test). In all cases a value of *P <*0.05 was considered statistically significant.

## Results

**Figure 1:**
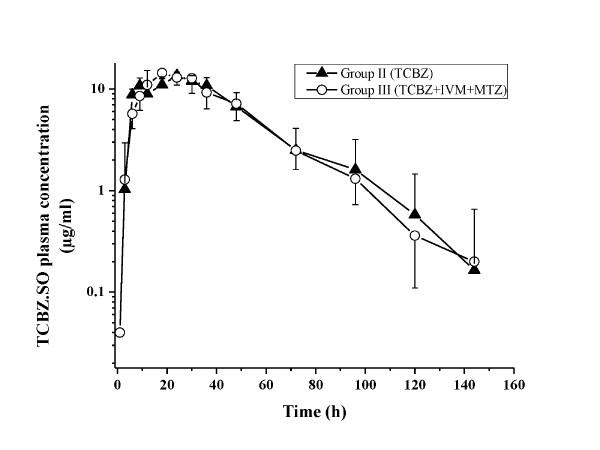
**TCBZ.SO plasma concentrations**. Comparative mean (±SD) plasma concentration profiles for triclabendazole sulphoxide (TCBZ.SO) measured after the administration of triclabendazole (TCBZ) either alone or co-administered with ivermectin (IVM) and methimazole (MTZ) to *Fasciola hepatica-*infected sheep.

**Figure 2:**
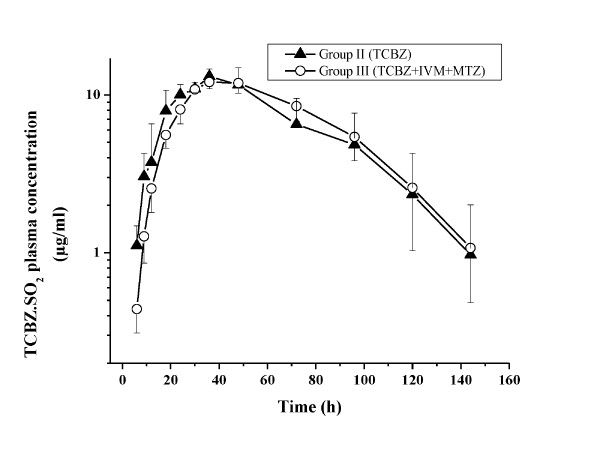
**TCBZ.SO_2 _plasma concentrations**. Comparative mean (±SD) plasma concentration profiles for triclabendazole sulphone (TCBZ.SO_2_) measured after the administration of triclabendazole (TCBZ) either alone or co-administered with ivermectin (IVM) and methimazole (MTZ) to *Fasciola hepatica-*infected sheep.

TCBZ.SO and TCBZ.SO_2 _were the only analytes recovered in plasma after the i.r. administration of TCBZ alone or co-administered with IVM and MTZ in TCBZ-resistant *F. hepatica*-infected sheep. High concentrations of both TCBZ metabolites were measured in plasma up to 144 h post-treatment (in both treated groups). The comparative mean (±SD) plasma concentration profiles of TCBZ.SO and TCBZ.SO_2 _obtained after the i.r. administration of TCBZ alone or co-administered with IVM and MTZ are shown in Figure 1 (TCBZ.SO) and Figure 2 (TCBZ.SO_2_). Table 1 summarizes the plasma pharmacokinetic parameters obtained for TCBZ.SO and TCBZ.SO_2 _after the i.r. administration of TCBZ alone or co-administered with IVM and MTZ to *F. hepatica*-infected sheep. The presence of IVM and MTZ did not affect the plasma disposition kinetics of TCBZ metabolites after the i.r. administration of TCBZ. The AUC value obtained for TCBZ.SO after TCBZ administration (653.9 ± 140.6 μg.h/ml) was similar to that obtained after TCBZ co-administered with IVM and MTZ (650.7 ± 122.8 μg.h/ml).

**Table 1:** Plasma pharmacokinetic parameters (mean ± SD) for triclabendazole sulphoxide (TCBZ.SO) and triclabendazole sulphone (TCBZ.SO_2_) obtained after the intraruminal (i.r.) administration of triclabendazole (TCBZ, 10 mg/kg, i.r.) alone or co-administered with ivermectin (IVM, 0.2 mg/kg, s.c.) and methimazole (MTZ, 1.5 mg/kg, i.m.) to *Fasciola hepatica-*infected sheep.

PHARMACOKINETIC PARAMETERS	TCBZ.SO		**TCBZ.SO**_**2**_	
	
	TCBZ alone	Combined treatment	TCBZ alone	Combined treatment
Cmax (μg/ml)	14.0 ± 0.85	15.6 ± 1.46	13.5 ± 1.68	12.3 ± 1.28
Tmax (h)	22.5 ± 7.55	24.0 ± 4.90	39.0 ± 6.00	42.0 ± 6.93
AUC_0-t_(μg.h/ml)	653.9 ± 140.6	650.7 ± 122.8	868.2 ± 217.6	893.7 ± 114.1
AUC_0-∞ _(μg.h/ml)	661.5 ± 148.5	657.1 ± 119.4	917.9 ± 270.6	945.6 ± 129.3
T1/2el (h)	17.5 ± 8.45	18.4 ± 5.82	26.8 ± 10.9	30.4 ± 9.30
MRT (h)	38.8 ± 10.5	39.1 ± 5.03	61.3 ± 17.2	67.6 ± 9.71
T1/2for (h)	6.85 ± 2.18	8.26 ± 1.22	12.3 ± 2.90	13.5 ± 1.93

**Cmax**: peak plasma concentration; **Tmax**: time to the Cmax; **AUC**_**0-t**_: Area under the plasma concentration vs. time curve from 0 to the detection time; **AUC**_**0-∞**_**(μg.h/ml): **Area under the plasma concentration vs. time curve extrapolated to infinity; **T1/2el**: elimination half-life; **MRT**: mean residence time (obtained by non-compartmental analysis of the data); **T1/2for**: formation half life.

Table 2 shows parasite counts and the clinical efficacy (%) of TCBZ against TCBZ-resistant *F. hepatica*, after its i.r. administration alone or co-administered with IVM and MTZ. Efficacy values of 56 and 38% were observed for TCBZ alone and for the combined treatment, respectively. No statistical differences (P > 0.05) were observed in fluke counts between treated groups and untreated control, which confirm the resistant status of the Sligo isolate.

**Table 2:** Individual and mean fluke counts and clinical efficacy (%) against triclabendazole (TCBZ)-resistant *Fasciola hepatica *obtained after the administration of TCBZ alone (10 mg/kg, i.r.) or co-administered with ivermectin (IVM, 0.2 mg/kg, s.c.) and methimazole (MTZ, 1.5 mg/kg, i.m.) to *Fasciola hepatica-*infected sheep.

	Untreated control	TCBZ alone	Combined treatment
	19	2	6
	11	12	9
	14	8	12
	9	5	6

**Arithmetic mean**	13.25	6.75	8.25
Efficacy*	-	**56%**	**38%**

* The efficacy was calculated using geometric means.

## Discussion

The use of drug combinations is becoming an alternative tool for therapeutic control of anthelmintic-resistant parasites. However, it is important to understand the potential pharmacokinetic/pharmacodynamic interactions between anthelmintic molecules, before drug combination formulations are developed to be introduced onto the pharmaceutical market.

Consistent with kinetic data previously obtained in sheep [27], TCBZ.SO and TCBZ.SO_2 _were the main metabolites recovered in plasma after the i.r. administration of TCBZ, which has been related to a first-pass oxidation occurring mainly in the liver. TCBZ.SO accounted for 42% of the total analytes found in sheep plasma after the i.r. administration of TCBZ. Both TCBZ metabolites were recovered in plasma for a period of 144 h post-treatment. The long persistence and high concentrations of TCBZ.SO and TCBZ.SO_2 _in sheep plasma compared with other BZD anthelmintics [28] are explained by the strong binding of both metabolites to plasma proteins [27]. This pharmacological property offers some advantage to TCBZ compared to other benzimidazole compounds in the activity against blood-feeding adult flukes.

Sulphoxidation and sulphonation are the main metabolic reactions involved in TCBZ hepatic biotransformation in sheep. A recent *in vitro *work showed that both mixed function oxidases, FMO and cytochrome P-450 (CYP), are involved in such metabolic reactions in sheep liver [18]. It was demonstrated that TCBZ sulphoxidative metabolism was reduced in the presence of the FMO inhibitor MTZ and also when the anthelmintic molecule was incubated in the presence of the CYP inhibitor piperonyl butoxide (PB) [18]. On the other hand, *in vivo *interference with the liver FMO-mediated and/or CYP-mediated metabolism has been shown to result in pronounced modifications of the pharmacokinetic behaviour of anthelmintically active BZDs metabolites. For example, in sheep, co-administration of oxfendazole with MTZ [29] increased the concentration of the active moieties (fenbendazole and oxfendazole) in the systemic circulation. Furthermore, MTZ and metyrapone, a potent inhibitor of the CYP system, improved the plasma availabilities of albendazole metabolites following the administration of the pro-BZD netobimin to sheep [30].

The pharmacokinetic interaction between albendazole and IVM in sheep was recently demonstrated. Higher albendazole sulphoxide plasma AUC was obtained after the i.r. administration of ABZ co-administered with IVM given s.c. to lambs [16]. Moreover, the co-administration of TCBZ and IVM by the i.v. route to sheep was correlated with higher peak plasma concentrations of TCBZ metabolites compared to those obtained following TCBZ alone [17]. The mechanism implicated in TCBZ-IVM interaction remains unclear. However, since both compounds have been described as Pgp substrates [8,9,14], it could be based on a drug-drug interaction via a transporter(s)-mediated drug efflux mechanism. Additionally, considering that IVM [31] as well as TCBZ and its metabolites [27] are strongly bound to plasma proteins, a drug binding displacement may occur when TCBZ and IVM are co-administered. Any interaction between IVM and MTZ has been described in the literature.

When TCBZ was administered by the i.r. route simultaneously with IVM (s.c.) and MTZ (i.m.) (current experiment), the plasma concentration profile of TCBZ.SO and TCBZ.SO_2 _were similar to that observed after the administration of TCBZ alone. In agreement with our results, the presence of MTZ did not modify the plasma pharmacokinetic behaviour of TCBZ metabolites in sheep [19], which indicates that the interaction between TCBZ and MTZ observed under *in vitro *conditions is not achieved *in vivo*. Furthermore, while IVM modifies the plasma pharmacokinetic behaviour of TCBZ metabolites after its simultaneous i.v. administration [17], the presence of IVM (after its s.c. administration) did not affect the plasma pharmacokinetic behaviour of TCBZ metabolites (after the i.r. administration of TCBZ). However, when we analyze the differences in IVM plasma concentrations according to the route of administration, we can observe that after its i.v. administration, IVM reaches an initial plasma concentration of 281.2 ± 32.6 ng/ml, which was significantly higher than its peak plasma concentration (21.3 ± 13.3 ng/ml) obtained after the s.c. administration at the same dose rate (Alvarez et al., 2008). It is clear that the route of administration may influence the drug concentration profiles over time at the different tissue/fluids. Thus, the potential drug-drug interaction may be influenced by the used route of administration of each compound

The WAAVP guidelines indicate that drug efficacy should be expressed as highly effective (over 98%), effective (90-98%), moderately effective (80-89%) or insufficiently active (less than 80%) [25]. According to this guideline, the results obtained in the present trial confirm the high level of resistance to TCBZ for the Sligo isolate, which has been described previously [22,23,32]. The experimental evidence, accumulated after different *ex vivo *experiments [5-7,15], demonstrate that altered drug efflux and enhanced metabolism may contribute to the development of resistance to TCBZ in *F. hepatica*. The co-incubation of TCBZ or TCBZ.SO with IVM results in higher drug accumulation into TCBZ-resistant *F. hepatica *compared to that observed after the incubation of TCBZ or TCBZ.SO alone [7]. Furthermore, the rate of sulphoxidative metabolism of TCBZ into TCBZ.SO was significantly higher (>40%) in TCBZ-resistant flukes [6]. This metabolic pathway described in the resistant flukes was significantly inhibited by MTZ [6]. From the results obtained in the present work, we can conclude that the co-administration of TCBZ with a Pgp substrate/inhibitor (IVM) and a metabolic inhibitor (MTZ) did not increase the clinical efficacy of TCBZ against TCBZ-resistant *F. hepatica *compared to the administration of TCBZ alone. This result may have two potential explanations: a) an alternative mechanism of TCBZ resistance may play a critical role under *in vivo *conditions, or b) the interaction between TCBZ-IVM-MTZ under our *in vivo *conditions does not achieve adequate magnitude at the level of the fluke to reverse TCBZ resistance. For example, the IVM concentration (1 μg/ml) used in the *ex vivo *experiments [7], is not achieved in bile after the s.c. administration of IVM (0.2 mg/kg) in sheep.

## Conclusions

In conclusion, the presence of IVM and MTZ did not affect the disposition kinetics of TCBZ and its metabolites. Thus, the combined drug treatment did not reverse the poor efficacy of TCBZ against TCBZ-resistant *F. hepatica*.

## Competing interests

The authors declare that they have no competing interests.

## Authors' contributions

LM and LC participate in the animal and analytical phase of the experiment and in writing the draft manuscript. LA and CL conceived the study, participated in its design and in the animal phase, and revised the draft version of the manuscript. LS and IF produced the metacercaries and revised the draft version of the manuscript. All authors have read and approved the final manuscript.
